# Sulfur dioxide exposure of mice induces peribronchiolar fibrosis—A defining feature of deployment-related constrictive bronchiolitis

**DOI:** 10.1371/journal.pone.0313992

**Published:** 2025-01-24

**Authors:** Seagal Teitz-Tennenbaum, Kayla N. Marinetti, Shayanki Lahiri, Khadijah Siddiqui, Celia Flory, Karinne Tennenbaum, Helen G. Hicks, Brian Song, Anutosh Ganguly, John J. Osterholzer

**Affiliations:** 1 Research Service and Pulmonary Section Medical Service, Veterans Affairs Ann Arbor Health System, Ann Arbor, Michigan, United States of America; 2 Division of Pulmonary and Critical Care Medicine, Department of Internal Medicine, University of Michigan, Ann Arbor, Michigan, United States of America; 3 Department of Surgery, University of Michigan, Ann Arbor, Michigan, United States of America; Alexandria University, EGYPT

## Abstract

Deployment-related constrictive bronchiolitis (DRCB) has emerged as a health concern in military personnel returning from Southwest Asia. Exposure to smoke from a fire at the Al-Mishraq sulfur enrichment facility and/or burn pits was reported by a subset of Veterans diagnosed with this disorder. DRCB is characterized by thickening and fibrosis of small airways (SA) in the lung, but whether these are related to toxin inhalation remains uncertain. The aim of this study was to determine whether sulfur dioxide (SO_2_) exposure can induce histopathological features of DRCB. C57BL/6J mice were exposed to 50 ± 5 ppm SO_2_ for one hour/day for five consecutive days. Lungs from exposed and unexposed mice were evaluated on day 5, 10, and 20. Lung sections were stained using hematoxylin and eosin, Masson’s trichrome, picrosirius red (PSR), and immunofluorescence for club cell secretory protein, acetylated-α-tubulin, and Ki67. Small airway wall thickness was determined by morphometric analysis and collagen content was quantified by measuring PSR fluorescence intensity. CurveAlign and CT-FIRE were used to enumerate collagen fibers and assess fibers’ width and length, respectively. Leukocyte subpopulations were quantified by flow cytometry analysis. This protocol of SO_2_ exposure of mice: 1) Triggered club cell proliferation and differentiation; 2) Increased SA wall thickness by inducing subepithelial collagen deposition; and 3) Increased width, length, and number, but not density, of collagen fibers within the wall of SA. 4) Induced no peribronchiolar inflammation or respiratory bronchiolitis. Collectively, these findings implicate club cell proliferation and differentiation in the profibrotic response to SO_2_ and identify this SO_2_ exposure as a potentially effective though imperfect model for studying SA fibrosis in DRCB.

## Introduction

Since the Persian Gulf War in 1990, more than 3.7 million United States military personnel (MP) have been deployed to Southwest Asia and Afghanistan [1]. Numerous studies have documented an increased incidence of persistent respiratory symptoms and increased prevalence of chronic lung disease diagnosed amongst Veterans previously deployed to this region [2–12]. While many cases can be attributed to well-defined pulmonary disorders such as asthma, a substantial percentage of previously deployed MP with exertional dyspnea and other respiratory complaints remain undiagnosed despite undergoing an extensive, non-invasive medical evaluation [13]. A prominent study by King et al [14] described the results of surgical lung biopsies performed on MP with unexplained exertional dyspnea and exercise intolerance. It identified histopathological evidence of constrictive bronchiolitis, defined as more than a 20% increase in small airway wall thickening, in 38 of 49 soldiers. Subsequent reports [15, 16] have corroborated these findings, and a recent study [17] identified peribronchiolar fibrosis as the defining feature of deployment-related constrictive bronchiolitis (DRCB). These studies also highlighted additional histopathologic abnormalities that may be detected in association with DRCB including peribronchiolar inflammation and respiratory bronchiolitis. The high percentage of patients with features of DRCB in these selected case series contrast with a case series review of lung biopsy tissue submitted to the Department of Veterans Affairs Joint Pathology Center in which the number of cases exhibiting specific features of constrictive bronchiolitis was low [18]. The uncertainty regarding the actual prevalence of DRCB may be attributed to the insensitivity of current non-invasive clinical tests to detect the condition and the infrequent use of invasive surgical lung biopsies. The spectrum of histopathologic abnormalities observed in DRCB suggests a multi-compartmental toxic lung injury and raises pressing questions regarding the etiology, pathophysiology, natural history, diagnosis, and treatment of deployment-related respiratory disease which includes DRCB [17, 19].

The specific cause of DRCB in MP returning from Southwest Asia and Afghanistan remains unknown, yet all MP diagnosed with DRCB report exposure to airborne hazards during deployment [14–16]. Many of them (46–74%) [14, 16] served in northern Iraq in the summer of 2003 and were exposed to smoke from the Al-Mishraq sulfur plant fire near Mosul. This fire, considered the largest non-volcanic sulfur dioxide (SO_2_) emission incident recorded to date, burned for almost a month producing dense clouds of SO_2_ estimated to total ~600 kilotons that spread over at least 100 square kilometers [20]. SO_2_ concentrations near the fire’s perimeter reached 52 ppm, whereas the US national air quality SO_2_ standard for a 24-hour period is 0.14 ppm [20]. In addition, a high percentage (42–63%) [14, 16] of MP diagnosed with DRCB were exposed to emissions from military base open air burn pits. These burn pits consisted of an array of solid waste from military operations that were ignited using jet fuel and continuously burned as a waste management strategy [21]. Emissions released by burn pits constitute a complex, variable mixture of chemicals and particulates. Potentially toxic substances identified in burn pit smoke include SO_2,_ polycyclic aromatic hydrocarbons, volatile organic compounds, and heavy metals [21]. The 2011 Institute of Medicine Report detailing the composition of burn pit smoke at Joint Base Balad acknowledged that concentrations of SO_2_ and other gaseous pollutants near open air burn pits have not been determined [21]. Reported exposures to desert dusts and sandstorms as well as combat smoke further broaden the scope of airborne hazards encountered by MP diagnosed with DRCB during deployment to Southwest Asia and Afghanistan [14, 16].

In response to growing concerns regarding the numerous potential airborne toxins present in deployment zones, a National Academies of Sciences, Engineering, and Medicine committee recently reviewed the available scientific and medical literature regarding respiratory health effects of exposure to airborne hazards encountered during service in the Southwest Asia theater of military operations. They concluded that, while there is an association between deployment and an increased incidence of respiratory symptoms, there is insufficient data to definitively link a specific exposure with any discrete medical condition including DRCB [1]. In this study, we used a murine model to evaluate whether exposure to SO_2_, a well-documented airborne hazard encountered during deployment to Southwest Asia, can induce histopathological features observed in MP diagnosed with DRCB. Our findings demonstrate that repetitive, 5-day SO_2_ exposure was sufficient to induce peribronchiolar fibrosis, the defining feature of DRCB. Moreover, epithelial injury triggering proliferation and differentiation of club cells preceded thickening of small airway walls due to collagen deposition potentially implicating this process in SA fibrogenesis.

## Materials and methods

### Mice

C57BL/6J female mice were obtained from the Jackson Laboratory (Bar Harbor, ME) and used for experiments at six to ten weeks of age. Triple transgenic Scgb1a1rtTA/tetOCre/R26:lacZ/DT-A (CC-DTA) mice described by Perl et al [22] have a reverse tetracycline transactivator gene driven by the club cell secretory protein (CCSP) promoter, a Cre recombinase gene under the control of a tet operator sequence, and a lox-P activated diphtheria toxin (DT) A gene. Ingestion of doxycycline by CC-DTA mice activates DTA expression only in club cells leading to targeted autonomous cell death. CC-DTA and single transgenic CRE littermate control mice were bred on site. All mice were housed under specific pathogen-free conditions in the Animal Care Facility at the Ann Arbor Veterans Affairs Health System. Animals were provided with food and water ad libitum, routine husbandry, standard housing, and 12-hour light/dark cycle.

### SO_2_ exposure

C57BL/6J mice were exposed to SO_2_ in cohorts of five using an exposure chamber installed and operated inside a certified fume hood. After placing mice in the transparent, plexiglass, air-tight exposure chamber (custom-designed by E-Z Systems, Bethlehem, PA), SO_2_ was slowly released from a 200 ppm in air 2000 psig tank (Airgas; Radnor, PA) controlled by a regulator set to 10 psi (without a flowmeter or filter) until the target level of 50 ± 5 ppm was reached inside the chamber. This target was selected based on reports of SO_2_ concentrations of 52 ppm near the perimeter of the fire at the Al-Mishraq sulfur enrichment facility guarded by U.S. MP in 2003 [20]. An extension tube mounted on the side wall of the exposure chamber promoted even gas distribution. An SO_2_ analyzer (Pro GasBadge by Industrial Scientific, Pittsburgh, PA) placed inside the chamber allowed continuous, real-time monitoring of SO_2_ levels throughout the exposure period. Another SO_2_ analyzer was placed in the fume hood outside the chamber to monitor for potential leaks in the exposure apparatus. At the end of the exposure period, SO_2_ was displaced from the chamber by allowing pressurized air to flow in. A regulated vacuum system connected to the exposure chamber facilitated SO_2_ and air flow and enabled constant adjustment of SO_2_ levels inside the chamber. A check (one-way) valve installed on a y-arm of the vacuum line ensured constant normal pressure within the chamber. A carbon dioxide analyzer (AmProbe, Seattle, WA) and an oxygen analyzer (Altair by MSA, Cranberry Township, PA) placed inside the chamber during SO_2_ exposure enabled continuous monitoring of these gas levels and adjustment of air flow to maintain levels within the normal range.

Mice were exposed to 50 ± 5 ppm SO_2_ for one hour a day for five consecutive days, on protocol day zero till four. Cohorts of age- weight- and gender-matched mice exposed to air served as control groups. Mice were sacrificed for lung harvest on protocol day 5, 10, and 20.

### Doxycycline exposure

Sustained club cell injury leading to murine constrictive bronchiolitis was induced by exposure of CC-DTA mice to doxycycline via their food (625 mg doxycycline/kg chow; Teklad, Envigo, Madison, WI) for 10 consecutive days, on protocol day zero till ten [22, 23]. Mice were sacrificed for lung harvest on protocol day 20.

### Histological evaluation and morphometric/quantitative analysis of lung sections

The lungs of mice were inflation-fixed in situ via the trachea with 10% neutral buffered formalin (NBF; Thermo Fisher Scientific, Waltham, MA). Lung lobes were then harvested, further fixed in 10% NBF, processed and embedded in paraffin. Four μm sections were stained with hematoxylin and eosin (H&E), Masson’s trichrome, picrosirius red (PSR), and for fluorescence immunohistochemistry according to standard laboratory procedures. The following primary antibodies were used: rabbit anti-club cell secretory protein (CCSP or uteroglobin, 1:100; Abcam, Cambridge, MA), mouse anti-acetyl-alpha tubulin (1:100; Sigma-Aldrich, St. Louis, MO), rat anti-Ki67 (1:100, Invitrogen, Waltham, MA). Secondary antibodies included: goat anti-rabbit-AF Plus 488, goat anti-mouse-AF Plus 647, and donkey anti-rat-AF Plus 647 (all at 1:200, Invitrogen). Sections were quenched and mounted using TrueVIEW autofluorescence quenching kit with DAPI (Vector Laboratories, Burlingame, CA). H&E-stained sections were imaged using a Zeiss Apotome microscope equipped with a Zeiss AxioCam MRm camera using the AxioVision software. Trichrome, PSR, and immunofluorescence-stained sections were imaged using a Keyence (Itasca, IL) BZ-X810 All-in-One fluorescence microscope.

Histological evaluation, morphometric measurements, and fluorescence intensity including collagen fiber analysis were performed on random lung sections (five lobes per lung section per mouse) of two mice per group. All lung lobes were scanned and pictures of ten randomly selected SA per lobe were photographed and analyzed to yield n = 100 SA. Small airways in the lungs of mice were defined as airways having an internal diameter of 45–400 μm. For one control mouse, only 4 lung lobes were available for analysis (n = 90 SA). In the CC-DTA model, all SA per lung lobe were analyzed (n = 60–79 SA).

To determine the extent of peribronchiolar fibrosis in the lungs of mice exposed to SO_2_, several approaches were employed:

1. To quantify small airway wall thickness, the area (in μm^2^) of subepithelial collagen deposition in trichrome-stained lung sections was determined by demarcating the basement membrane and the outer edge of the airway adventitia. For each small airway analyzed, the area of collagen deposition was normalized to the length of the subepithelial basement membrane (in μm). Morphometric analysis was performed using ImageJ2 software (National Institutes of Health, Bethesda, MD).
2. To quantify collagen content within the wall of SA, fluorescence intensity of PSR in small airway walls of PSR-stained lung sections was measured using ImageJ2 software. Each small airway analyzed was designated as a region of interest (ROI) and background fluorescence was subtracted. PSR fluorescence intensity was then normalized to the length of the subepithelial basement membrane. The latter was measured in images acquired using the phase contrast mode.
3. To quantify the number of collagen fibers in the wall of SA, fluorescent microscope images of SA from PSR-stained lung sections were analyzed using CurveAlign [24, 25] fiber detection software (an open-source MATLAB based tool). Collagen density in the wall of SA was calculated by dividing the number of collagen fibers by the area (in mm^2^) of subepithelial collagen deposition. The latter was measured in images acquired using the phase contrast mode.
4. To quantify the mean width and length of collagen fibers in the wall of SA, fluorescent microscope images of SA from PSR-stained lung sections were analyzed using CT-FIRE [25–27] fiber detection software (an open-source MATLAB based tool).

To determine the extent of small airway epithelial injury in the lungs of mice exposed to SO_2_, lung sections were stained using immunofluorescence for CCSP (club cell marker) and acetylated-α-tubulin (ciliated cell marker). Fluorescence intensity of these markers in SA was quantified using ImageJ2 software as described above for PSR and normalized to the length of the subepithelial basement membrane. To determine the extent of club cell proliferation in the epithelium of SA, lung sections were stained using immunofluorescence for the proliferation marker Ki67, CCSP, and DAPI. The percentage of proliferating club cells was calculated using the following formula: [(Ki67^+^DAPI^+^CCSP^+^/DAPI^+^CCSP^+^) * 100]. Cells were enumerated using ImageJ2 software. In lung sections immune-stained for Ki67, some lobes had less than ten SA yielding n = 89–98 SA.

For context and comparative purposes, histopathologic findings from three former military personnel diagnosed with deployment-related respiratory disease are provided. One of these individuals was exposed to the fire at the Mosul sulfur enrichment facility, whereas the other two were not. All three reported exposures to military burn pits. Human surgical lung biopsies were formalin fixed and paraffin embedded. Sections were stained with H&E and Masson’s trichrome. Immunohistochemistry for CD68 (monocyte/macrophage marker) was performed using a diaminobenzidine peroxidase reaction following clinical laboratory protocols at the VA Ann Arbor Health System. The diagnosis of DRCB was confirmed by a histopathological review at Vanderbilt University Medical Center.

### Harvest and processing of lungs for flow cytometry analysis

For flow cytometric analysis, lungs were perfused in situ via the right heart using 10 ml of PBS to clear pulmonary vessels. Lung lobes were then harvested, minced, and placed in digestion buffer (5 ml/lung) containing 5% complete medium [CM; RPMI medium 1640, fetal bovine serum (5%), penicillin-streptomycin (1%), MEM Non-Essential Amino Acids Solution (1%), and sodium pyruvate (1%); all from Gibco by Life Technologies], deoxyribonuclease I (250 Kunitz units/lung; Sigma-Aldrich, St. Louis, MO), and collagenase type I (0.1%; Gibco by Life Technologies). Lungs were mechanically homogenized twice using a gentleMacs dissociator (Miltenyi Biotec, Auborn, CA), and enzymatically digested between homogenization cycles at 37°C for 35 minutes on a rocker. After erythrocyte lysis by ACK (KD Medical, Columbia, MD), cells were washed and filtered over a 100 μm cell strainer. Dead cells were removed by centrifugation over a Percoll (Sigma-Aldrich) gradient. Viable, lung-derived cells in each sample were enumerated in the presence of Trypan Blue (Gibco by Life Technologies) using a hemocytometer. Each lung was processed and analyzed individually.

### Cell staining and flow cytometry analysis

Cells were stained with a fixable viability dye (Zombie aqua; BioLegend, San Diego, CA) following the manufacturer’s protocol. After blocking Fc receptors using anti-CD16/32 antibody (clone 93, BioLegend), cells were stained for cell surface markers using fluorochrome-conjugated antibodies listed in S1 Table, and then fixed with 2% formaldehyde (ThermoFisher Scientific) in PBS. Data were acquired using an LSRFortessa flow cytometer (BD Biosciences) and analyzed using FlowJo software v.10.7.1 (Treestar, Ashland, Oregon). One hundred thousand events in the CD45^+^ gate were acquired per lung sample. To determine the number of cells per each population of interest per lung, the corresponding percentage was multiplied by the total number of viable CD45^+^ cells in that lung sample. The latter value was calculated for each sample as the product of the percentage of viable CD45^+^ cells and the original hemocytometer count of total viable cells identified within that sample.

### Statistical analysis

Data in graphs are presented as mean ± standard error of the mean (SEM). Data were evaluated by unpaired, two-tailed t-test, corrected for multiple comparisons using the Holm-Sidak method or one-way analysis of variance (ANOVA) followed by Tukey’s multiple comparisons test. Statistical analysis was performed using Graph-Pad Prism software version 9. P values < 0.05 were considered statistically significant.

### Ethics

This study was carried out in accordance with the recommendations in the Guide for the Care and Use of Laboratory Animals of the National Institutes of Health. The animal protocol (Protocol Number: 1597355) was approved by the Institutional Animal Care and Use Committee and by the Biosafety Committee at the Veterans Affairs Ann Arbor Health System, Ann Arbor, Michigan. Deidentified images of human histological sections were obtained from the VA Ann Arbor Health System for representative purposes. The use of these images was deemed exempt from ethics review by the chair of the IRB Committee at the VA Ann Arbor Health System.

## Results

### SO_2_ exposure of mice increases small airway wall thickness by inducing peribronchiolar fibrosis recapitulating DRCB

**Fig 1:**
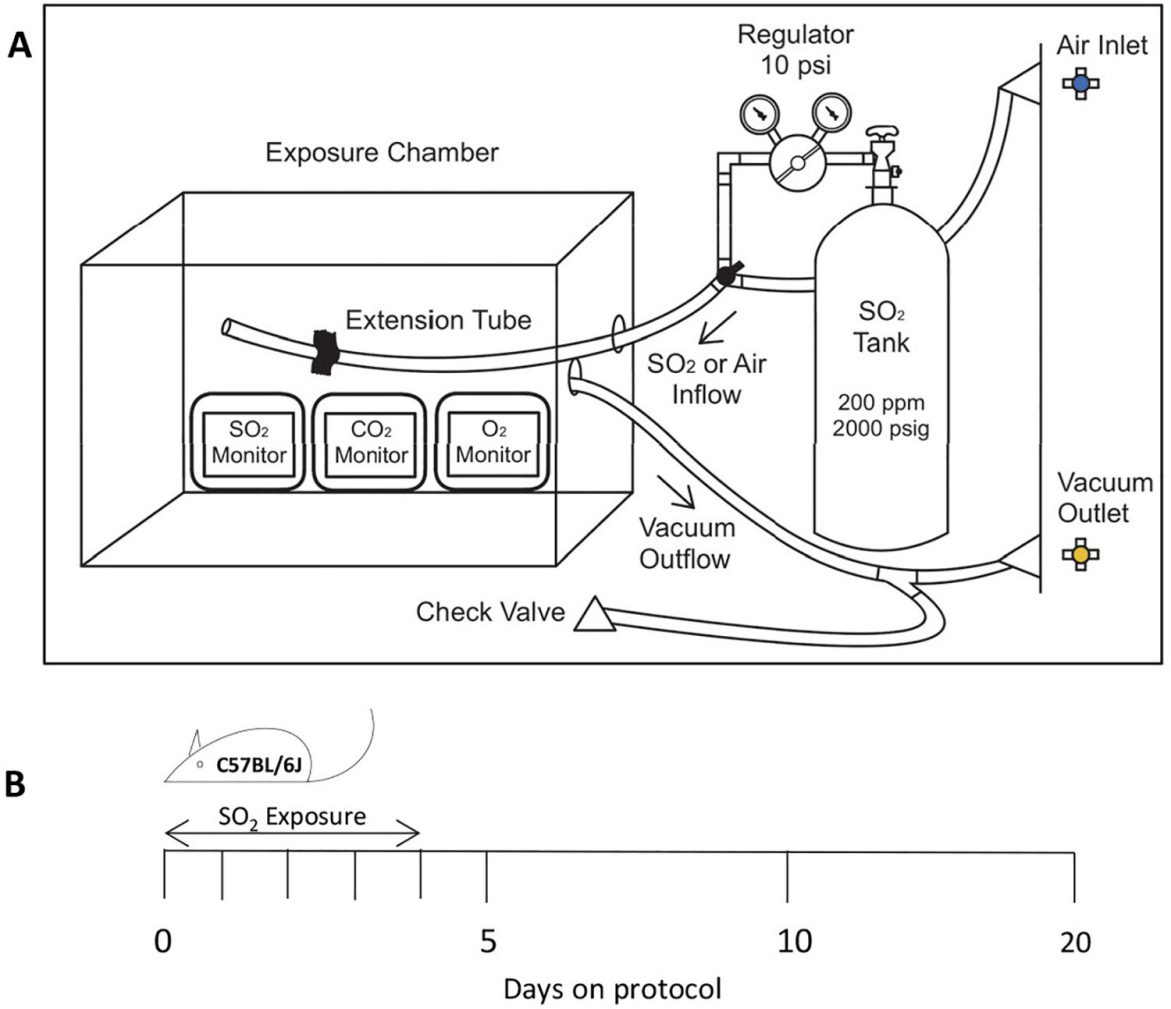
Sulfur dioxide (SO_2_) exposure chamber and exposure protocol. (A) Diagram of SO_2_ exposure chamber installed and operated inside a fume hood. (B) Schematic representation of SO_2_ exposure protocol. Cohorts of C57BL/6J mice were exposed to 50 ± 5 ppm SO_2_ in air for one hour a day for five consecutive days on protocol day 0–4. Control mice were not exposed to SO_2_. Lungs were harvested on protocol day 5, 10, and 20 for examination.

To investigate whether exposure to SO_2_ can induce histopathological features of DRCB, we engineered an exposure chamber as described in the Materials and Methods section and shown in Fig 1A. Cohorts of mice were exposed to 50 ± 5 ppm SO_2_ for one hour a day for five consecutive days, on protocol day zero till four (Fig 1B). Mice not exposed to SO_2_ (exposed to air) served as negative control groups. Mice exposed to SO_2_ under this regimen displayed no signs of distress, neither during the exposure periods nor throughout the duration of the experimental protocol, and no statistically significant weight loss versus control mice was observed.

**Fig 2:**
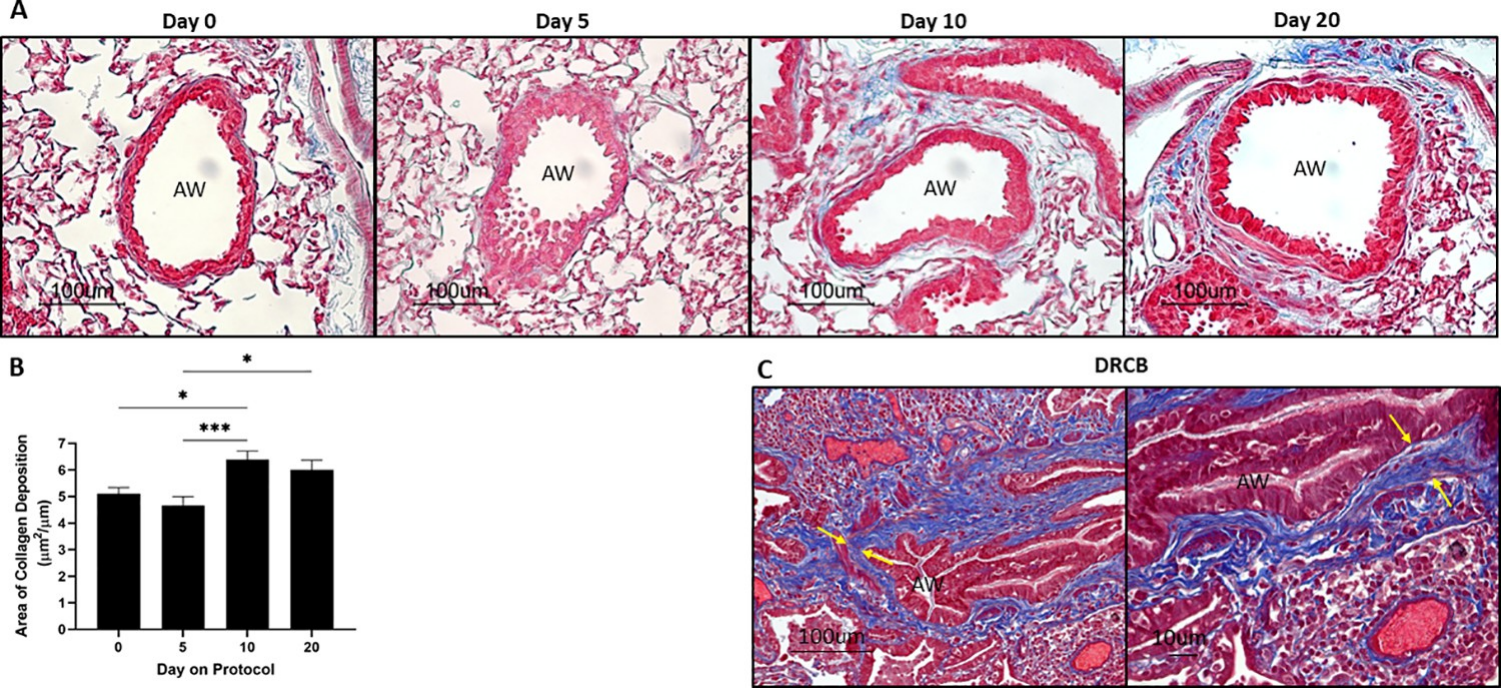
SO_2_ exposure increases small airway (AW) wall thickness. (A) Representative images of SA from Masson’s trichrome-stained lung sections of mice at the designated protocol time points. Day 0 denotes control, unexposed mice. Note blue stain, depicting collagen, surrounding SA at day 10 and 20. Scale bars: 100 μm. (B) Morphometric analysis of small airway wall thickness in lung sections from mice at the indicated protocol time points. The area, determined by demarcating the basement membrane and the outer edge of the airway adventitia, was normalized to the length of the subepithelial basement membrane. Data represent mean ± SEM of 90–100 SA; ten randomly selected SA per lung lobe, 4–5 lobes per mouse, two mice per time point. * P < 0.05, *** P < 0.001; one-way ANOVA followed by Tukey’s multiple comparisons test. (C) Representative images of a small airway from Masson’s trichrome-stained sections of a surgical lung biopsy of a Veteran diagnosed with DRCB. Note thickening of the airway wall due to subepithelial collagen deposition marked by yellow arrows. Scale bars: 100 μm (Left), 10 μm (Right).

The hallmark of DRCB is subepithelial fibrosis of SA in the lung [17]. This defining histopathological feature translates to increased thickness of the wall of SA due to collagen deposition. To evaluate the effect of SO_2_ exposure on small airway (defined as 45–400 μm in internal diameter) wall thickness in lungs of mice, Masson’s trichrome-stained lung sections of unexposed (day 0) and SO_2_ exposed mice at protocol day 5, 10, and 20 were examined. Representative images of SA from these sections are shown in Fig 2A. Enhanced deposition of collagen, evident as blue stain, in the wall of SA was observed at protocol day 10 and 20 versus day 0 and 5. The area (in μm^2^) of subepithelial collagen deposition in the wall of SA was quantified using morphometric measurements and normalized to the length (in μm) of the basement membrane. As shown in Fig 2B, normalized area of collagen deposition in the wall of SA was increased up to 25% at day 10 versus day 0 and 5 and at day 20 versus day 5.

To allow comparison of these findings with features described in DRCB, Fig 2C shows representative images of a small airway from Masson’s trichrome-stained sections of a surgical lung biopsy obtained from a Veteran evaluated and diagnosed with DRCB at our institution. In the left (lower magnification) and right (higher magnification) panels of Fig 2C, prominent thickening of the small airway wall due to subepithelial collagen deposition is evident consistent with previous reports of DRCB [14–16]. Additional images demonstrating peribronchiolar fibrosis are shown in S1 Fig and were obtained from surgical lung biopsies of two other Veterans heavily exposed to airborne toxins and diagnosed with deployment-related respiratory disease at our institution. Altogether, our results demonstrate that SO_2_ exposure of mice increases small airway wall thickness by promoting intramural collagen deposition in a manner comparable to that detected in DRCB.

### SO_2_ exposure of mice increases collagen content in the wall of small airways resembling sustained club cell injury-induced murine constrictive bronchiolitis

**Fig 3:**
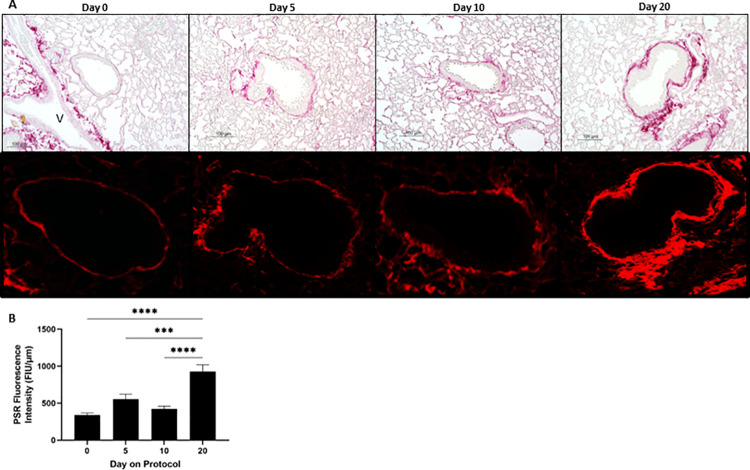
SO_2_ exposure increases collagen content in the wall of small airways. (A) Representative images of SA from picrosirius red (PSR)-stained lung sections of mice at the designated protocol time points. Day 0 denotes control, unexposed mice. Upper panels, phase-contrast microscopy; Lower panels, fluorescence microscopy; blood vessel, V. Note that the fine red stain, depicting collagen, surrounding small airways at day 0, 5, and 10 turns thick and coarse at day 20. Scale bars: 100 μm. (B) Subepithelial collagen content in the walls of SA of mice at the indicated protocol time points was quantified by measuring PSR fluorescence intensity in the wall of SA in PSR-stained lung sections (lower panels of A) and reported as fluorescence intensity units (FIU) normalized to the length of the subepithelial basement membrane (measured in upper panels of A) as detailed in the Materials and methods section. Data represent mean ± SEM of 90–100 SA; ten randomly selected SA per lung lobe, 4–5 lobes per mouse, two mice per time point. *** P < 0.001, **** P < 0.0001; one-way ANOVA followed by Tukey’s multiple comparisons test.

To validate and extend the data presented in Fig 2B, collagen content in the wall of SA was quantified by measuring PSR fluorescence intensity in fluorescent microscopy images of SA from PSR-stained lung sections. The upper panel of Fig 3A shows representative phase-contrast microscopy images of SA from PSR-stained lung sections of unexposed (day 0) and SO_2_ exposed mice at protocol day 5, 10, and 20. At baseline (day 0), a narrow region of collagen deposition identified by red staining was observed surrounding SA. (Note that the heavy red staining in day 0 panel is localized to the wall of a blood vessel.) Beginning on protocol day 5, an increase in collagen deposition beneath the small airway epithelium was identified in some airways and became much more prominent throughout the lung at protocol day 20. The lower panel of Fig 3A shows the same airways viewed using fluorescent microscopy which further enhances the visualization of increased subepithelial collagen deposition at protocol day 20. This increase in subepithelial collagen deposition was quantified and demonstrated that PSR fluorescence intensity within the wall of SA normalized to the length of the subepithelial basement membrane was substantially higher, up to 2.7-fold, at day 20 versus day 0, 5, and 10 (Fig 3B).

**Fig 4:**
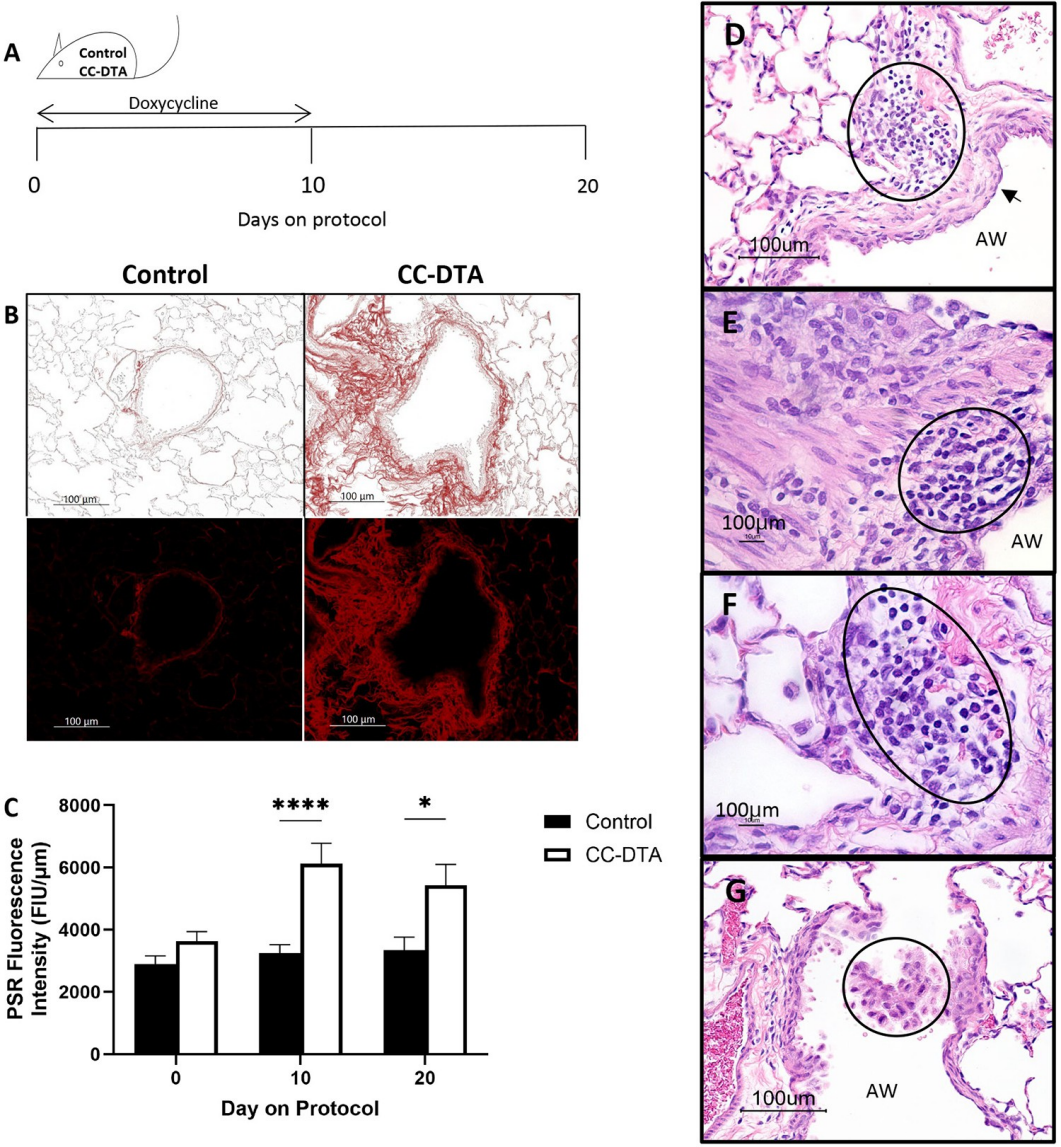
Sustained club cell injury increases collagen content in the wall of small airways and induces peribronchiolar inflammation. (A) Schematic representation of doxycycline exposure protocol. Cohorts of littermate control and CC-DTA mice were exposed to doxycycline in chow for ten consecutive days on protocol day 0–10. Lungs were harvested on protocol day 10, and 20 for examination. (B) Representative phase-contrast (upper panels) and fluorescent (lower panels) microscopy images of SA from PSR-stained lung sections of a control (Left) and CC-DTA (Right) mice exposed to doxycycline for 10 days and evaluated at protocol day 20. Note prominent red staining surrounding small airway of CC-DTA, but not control, mouse. Scale bars: 100 μm. (C) Subepithelial collagen content in the walls of SA from doxycycline-exposed control and CC-DTA mice at the indicated protocol time points (day 0 denotes unexposed mice) was quantified by measuring PSR fluorescence intensity in PSR-stained lung sections and reported as fluorescence intensity units (FIU) normalized to the length of the subepithelial basement membrane. Data represent mean ± SEM of 60–79 SA; all SA per lung lobe, five lobes per mouse, two mice per time point. * P < 0.05, **** P < 0.0001; unpaired t-test corrected for multiple comparisons using the Holm-Sidak method. (D-G) Representative images of small airways (AW) from H&E-stained lung sections of CC-DTA mice exposed to doxycycline for 10 days and evaluated at protocol day 20. Note features of DRCB such as squamous epithelial metaplasia (D, marked by an arrow), peribronchiolar mononuclear cell infiltrates (D, E, and F, marked by an oval), and clusters of intraluminal cells resembling enlarge, foamy macrophages (G, marked by a circle). Scale bars: 100 μm.

CC-DTA are inducible transgenic mice that in response to doxycycline exposure undergo targeted club cell death [22]. Club cells, local progenitors lining the SA, are critical for repairing the epithelium after exposure to various airborne toxins [28–30]. Sustained club cell injury, induced by ten consecutive days of doxycycline administration, has been shown to cause peribronchiolar fibrosis [22, 23]. Moreover, using comparative histopathological analysis, we have reported that sustained club cell injury is sufficient to recapitulate key features of DRCB [23]. To facilitate comparison of the findings described in Fig 3 with this established murine model of DRCB, Fig 4 shows representative phase-contrast (Fig 4B, upper panels) and fluorescent (lower panels) microscopy images of SA from PSR-stained lung sections of doxycycline-exposed control (left panels) and CC-DTA (right panels) mice at protocol day 20 (Fig 4A). Small airways of doxycycline-exposed control mice displayed minimal subepithelial collagen deposition (Fig 4B, left panels) similar to those observed in C57BL/6J mice not exposed to SO_2_ (Fig 3A, left panels). In contrast, SA of doxycycline-exposed CC-DTA mice with sustained club cell injury exhibited prominent subepithelial collagen deposition (Fig 4B, right panels) comparable to that detected in SO_2_-exposed mice on protocol day 20 (Fig 3A, right panels). Quantification of normalized PSR fluorescence intensity within the wall of SA revealed an increase of 89% and 62% on protocol day 10 and 20, respectively, in doxycycline-exposed CC-DTA versus control mice (Fig 4C). These results are in accordance with prior morphometric-based studies [22] and thus validate this methodology.

**Fig 5:**
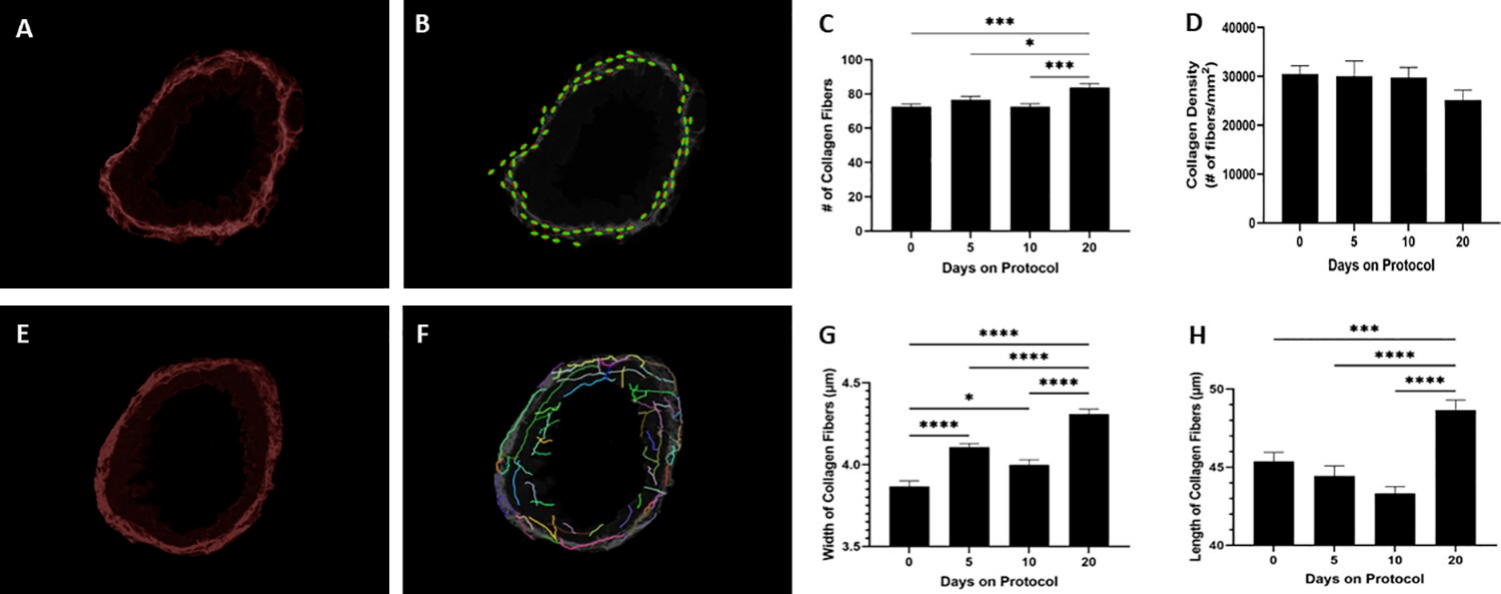
SO_2_ exposure increases the width, length, and number, but not density, of collagen fibers in the wall of small airways. (A) A representative fluorescence microscopy image of a small airway from PSR-stained lung sections of mice at protocol day 20 analyzed for number of collagen fibers using CurveAlign. (B) CurveAlign-generated schematic representation of the number of collagen fibers identified in panel A. (C) The number of collagen fibers in the wall of SA in PSR-stained lung sections of mice at the indicated protocol time points was quantified using CurveAlign. (D) The density of collagen fibers in the wall of SA in PSR-stained lung sections of mice at the indicated protocol time points was calculated by dividing the number of fibers by the area of collagen deposition. (E) A representative fluorescence microscopy image of a small airway from PSR-stained lung sections of mice at protocol day 20 analyzed for mean width and length of collagen fibers using CT-FIRE. (F) CT-FIRE-generated schematic representation of the width and length of collagen fibers identified in panel E. (G and H) The mean width (G) and length (H) of collagen fibers in the wall of SA in PSR-stained lung sections of mice at the indicated protocol time points was quantified using CT-FIRE. (C, D, G, and H) Data represent mean ± SEM of 90–100 SA; ten randomly selected SA per lung lobe, 4–5 lobes per mouse, and two mice per time point. * P < 0.05, *** P < 0.001, **** P < 0.0001; one-way ANOVA followed by Tukey’s multiple comparisons test. In panel D, no statistically significant differences between the groups were observed.

To further characterize collagen deposition in the wall of SA in response to SO_2_ exposure, the number of collagen fibers per small airway wall was quantified using CurveAlign analysis of fluorescent microscope images of SA from PSR-stained lung sections (Fig 5A and 5B). Increased number of collagen fibers per small airway wall was detected in lungs of mice at protocol day 20 versus day 0, 5, and 10 (Fig 5C). No statistically significant differences in collagen density, defined as the number of collagen fibers per small airway wall divided by the small airway wall area, were observed amongst the four groups of mice (Fig 5D). The mean width and length of collagen fibers within the wall of SA was determined using CT-FIRE analysis of fluorescent microscope images of SA from PSR-stained lung sections (Fig 5E and 5F). The mean width of collagen fibers within the wall of SA increased gradually post SO_2_ exposure and peaked at protocol day 20 (Fig 5G). Interestingly, the mean length of collagen fibers within the wall of SA increased only at protocol day 20 (Fig 5H). These findings suggest that SO_2_ exposure promotes airway wall thickening (Fig 2B) due to enhanced collagen deposition (Fig 3B) by increasing the number, width, and length of collagen fibers beneath the airway epithelium. Collectively, these data strongly indicate that exposure of mice to SO_2_ leads to the development of peribronchiolar fibrosis resembling that observed in sustained club cell injury-induced murine constrictive bronchiolitis.

### SO_2_ exposure of mice induces no peribronchiolar inflammation

**Fig 6:**
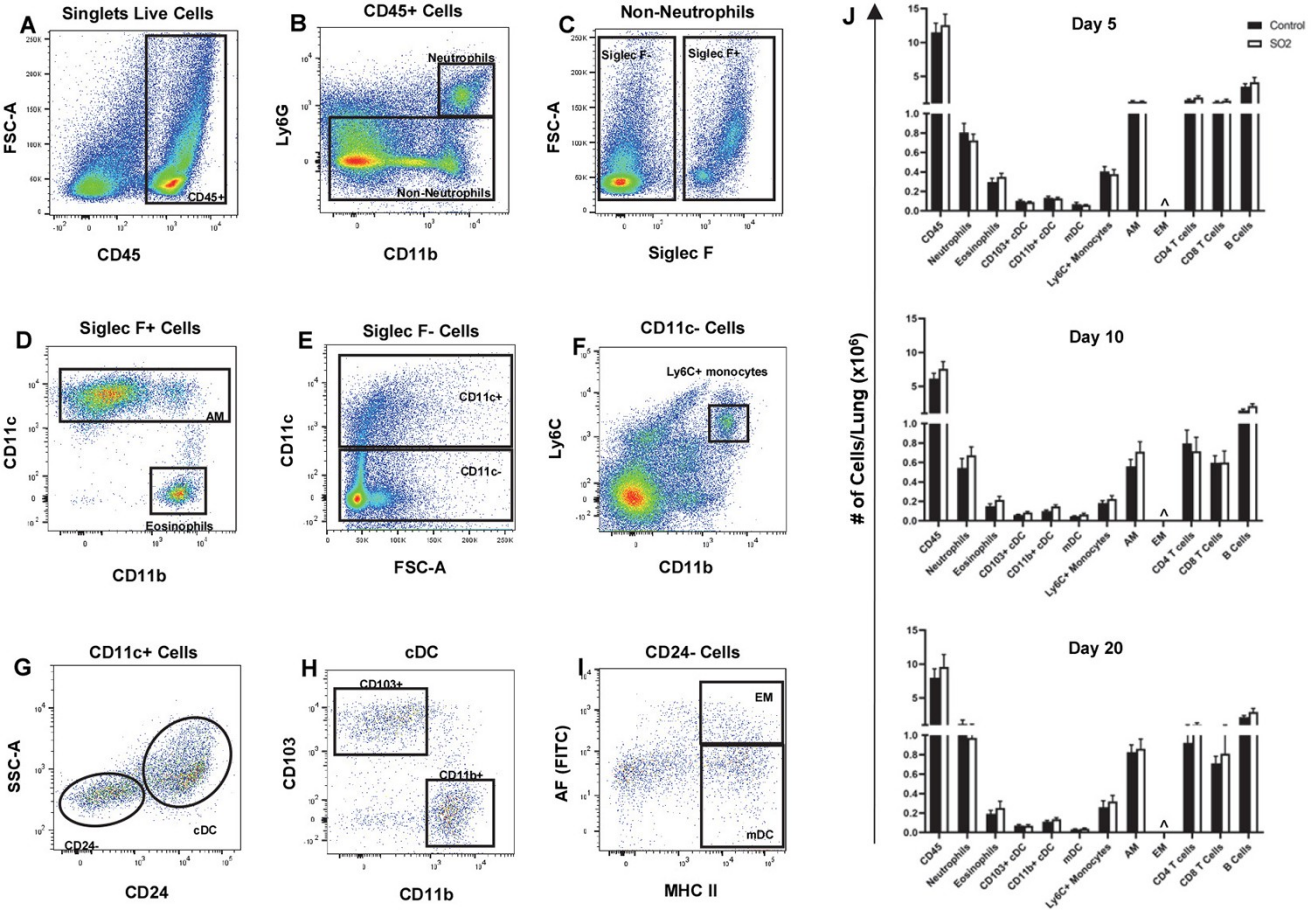
SO_2_ exposure induces no accumulation of leukocytes in the lung. (A-I) Gating strategy for identifying lung myeloid subsets in control and SO_2_-exposed mice. Lung-derived single cell suspensions obtained from control and SO_2_-exposed mice were stained using fluorochrome-conjugated antibodies targeting CD45, Ly6G, CD11b, Siglec F, CD11c, Ly6C, CD24, CD103, and MHC class II and analyzed by flow cytometry. Representative dot plots from a single mouse exposed to SO_2_ and evaluated on protocol day 10 are shown. After excluding doublets, debris, and dead cells, CD45^+^ white blood cells were identified (A). Neutrophils were detected based on expression of Ly6G and CD11b (B). Non-neutrophils, Siglec F^+^ cells (C) were depicted on CD11b versus CD11c plots to identify eosinophils and alveolar macrophages (AM, D). Ly6C^+^ monocytes were defined as non-neutrophils, Siglec F^-^CD11c^-^CD11b^+^Ly6C^+^ cells (E and F). Conventional DC (cDC) were identified as non-neutrophils, Siglec F^-^CD11c^+^CD24^+^ cells (G) and further classified based on expression of CD103 or CD11b (H). CD24^-^ cells within the CD11c^+^ gate were depicted on MHC class II versus autofluorescence (detected in the FITC channel) plots to identify exudate macrophages (EM) and monocyte-derived DC (mDC, I). FSC, forward scatter; SSC, side scatter; A, area, DC, dendritic cells, MHC, major histocompatibility complex. (J) Total numbers of leukocytes (CD45^+^ cells) and each indicated leukocyte subset per SO_2_ exposed and control mouse lung at protocol day 5 (upper), 10 (middle), and 20 (lower)were determined. Data represent mean ± SEM of five mice. ^ < 0.004 x 10^6^/lung for both control and SO^2^-exposed mice, bars are too low to be depicted. No statistically significant differences between SO_2_ exposed (white bars) versus control (black bars) mice were detected, unpaired t-test corrected for multiple comparisons using the Holm-Sidak method.

In addition to peribronchiolar fibrosis, many MP diagnosed with DRCB exhibit histological evidence of chronic small airway inflammation consisting of peribronchiolar immune infiltrates and/or respiratory bronchiolitis, defined as clusters of large, foamy macrophages located in the lumen of SA [14–16]. To examine whether exposure to SO_2_ triggers an inflammatory response in the lung, the total number of leukocytes (CD45^+^ cells) and various leukocyte subsets in lungs of mice unexposed and exposed to SO_2_ was quantified using flow cytometry analysis (Fig 6). Lung myeloid subpopulations were identified using an established gating strategy [23, 31] shown in Fig 6A–6I. Note that each time point was evaluated independently which facilitates comparison between SO_2_-exposed and control mice at each time point but limits comparisons of leukocyte numbers between SO_2_-exposed mice at different time points. At protocol day 5, 10, and 20, no statistically significant differences in the number of lung leukocytes or leukocyte subsets between SO_2_ exposed and unexposed mice were detected (Fig 6J). To determine whether exposure to SO_2_ modulates pulmonary and/or systemic production of inflammatory, regulatory, and/or profibrotic cytokines, cytokine levels in bronchoalveolar lavage fluid (BALF) and serum samples were quantified using a cytometric bead array (LEGENDplex, BioLegend, San Diego, CA) on protocol day 0 (unexposed mice), 10, and 20. Comparison of cytokine concentrations in BALF and serum samples at protocol day 10 and 20 versus day 0 (unexposed mice) revealed either undetectable levels or no statistically significant differences in tumor necrosis factor-alpha, IL-6, IL-12p70, IL-2, IL-4, IL-10, IL-13, interferon gamma, and free active transforming growth factor beta 1. These findings indicate that this SO_2_ exposure protocol induces no detectable accumulation of white blood cells and no apparent alteration in inflammatory or pro-fibrotic cytokines in the lungs of mice using the described techniques at the time points evaluated.

**Fig 7:**
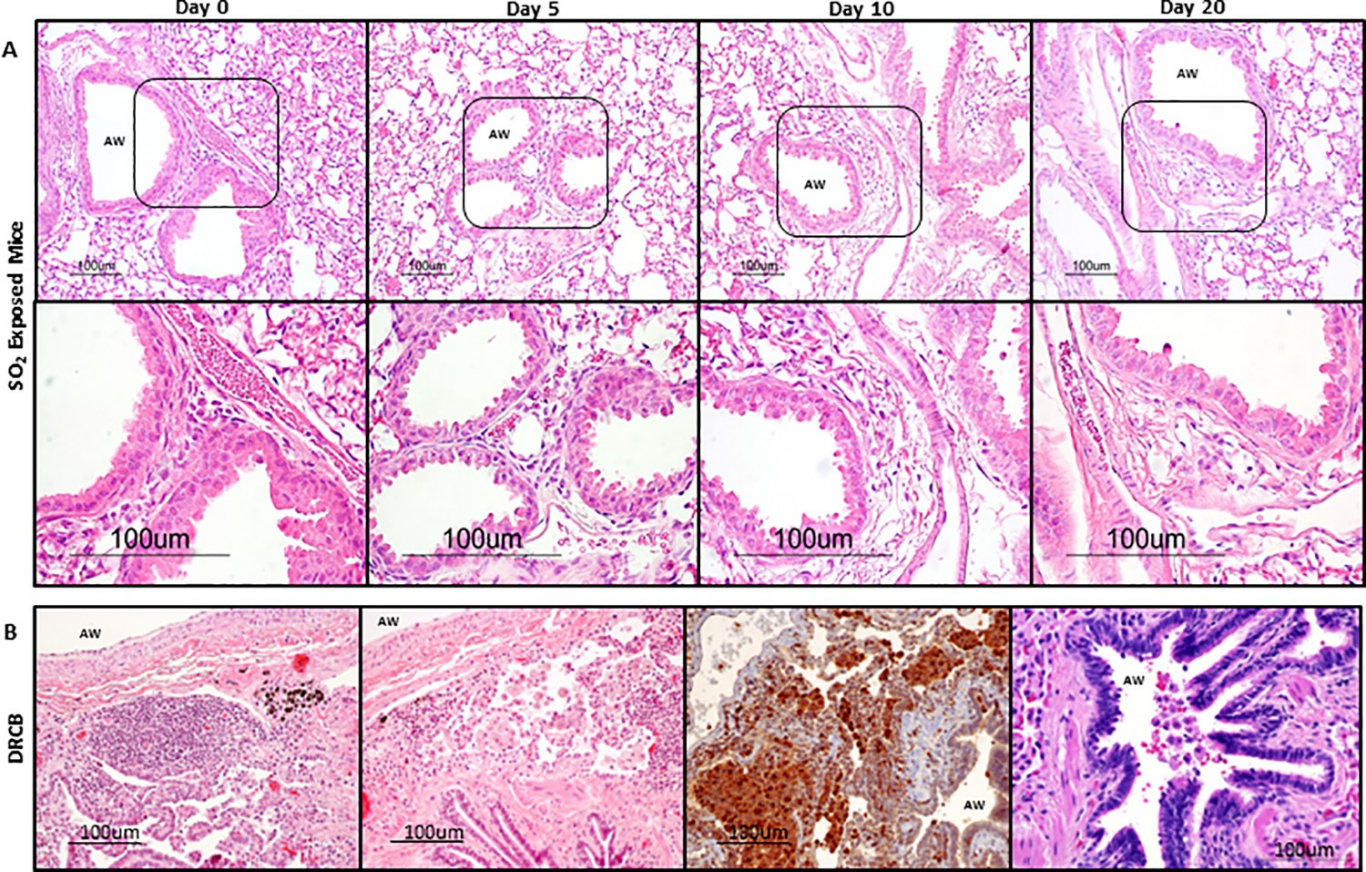
SO_2_ exposure induces no discernable peribronchiolar inflammation. (A) Representative images of SA from hematoxylin and eosin (H&E)-stained lung sections of mice at the designated protocol time points. Day 0 denotes control, unexposed mice. Note the presence of sparse, loose clusters of immune cells (marked by inserts in the upper panels) next to SA and blood vessels in both control and SO_2_ exposed mice, regardless of protocol time point. Lower panels show higher magnification of inserts. (B) Representative images of SA from lung sections of a Veteran diagnosed with DRCB. Left, middle left, and right panels were stained with H&E; middle right panel was stained for CD68 using immunohistochemistry (brown stain depicts monocytes and macrophages). Note peribronchiolar pigment deposition (left panel), peribronchiolar mononuclear cell infiltrates (left and middle left panels), peribronchiolar collections of CD68^+^ cells (middle right panel), and clusters of intraluminal cells resembling big, foamy macrophages (right panel).

Since assessment of whole lung leukocytes and cytokine levels might not be sensitive enough to detect a local immune response confined to the SA, we next evaluated H&E-stained lung sections to examine whether SO_2_ exposure elicits focal features of small airway inflammation. Scarce, loose clusters of mostly mononuclear cells were detected in the vicinity of SA and appeared comparable in SO_2_ exposed versus unexposed mice (Fig 7A). In contrast, representative images of SA from H&E-stained sections of a surgical lung biopsy obtained from the Veteran diagnosed with DRCB (as described in Fig 2C) displayed prominent peribronchiolar mononuclear cell infiltrates (Fig 7B, left and middle left panel) and collections of intraluminal large, foamy cells resembling macrophages (Fig 7B, right panel). Immunohistochemistry for CD68 confirmed that a monocyte-macrophage lineage dominated these peribronchiolar immune infiltrates (brown stain in Fig 7B, middle right panel). Similar histopathological findings detected in lung biopsies of two additional Veterans heavily exposed to airborne toxins and diagnosed with deployment-related respiratory disease at our institution are shown in S3 Fig and are in accordance with previous reports [14–16]. Additional histopathological features commonly described in DRCB [14–16] and shown in Fig 7B include squamous epithelial metaplasia (left and middle left panels) and peribronchiolar pigment deposition (left panel). Neither of these features were observed in SO_2_ exposed mice at the time points evaluated.

The absence of small airway inflammation in this model of SO_2_ exposure also contrasts with observations made using the sustained club cell injury-induced constrictive bronchiolitis mouse model. As previously reported [23], CC-DTA mice at protocol day 20 (Fig 4A) developed peribronchiolar fibrosis (Fig 4B and 4C) associated with squamous epithelial metaplasia (Fig 4D), mononuclear cell infiltrates in the vicinity of SA (Fig 4D–4F), and respiratory bronchiolitis (Fig 4G), thus recapitulating many of the inflammatory features identified in MP with DRCB. In concert, our findings show that mice exposed to SO_2_, using this specific protocol, developed peribronchiolar fibrosis but displayed no additional inflammatory histological abnormalities linked to DRCB.

#### SO_2_ exposure of mice induces club cell proliferation and differentiation indicating epithelial injury

**Fig 8:**
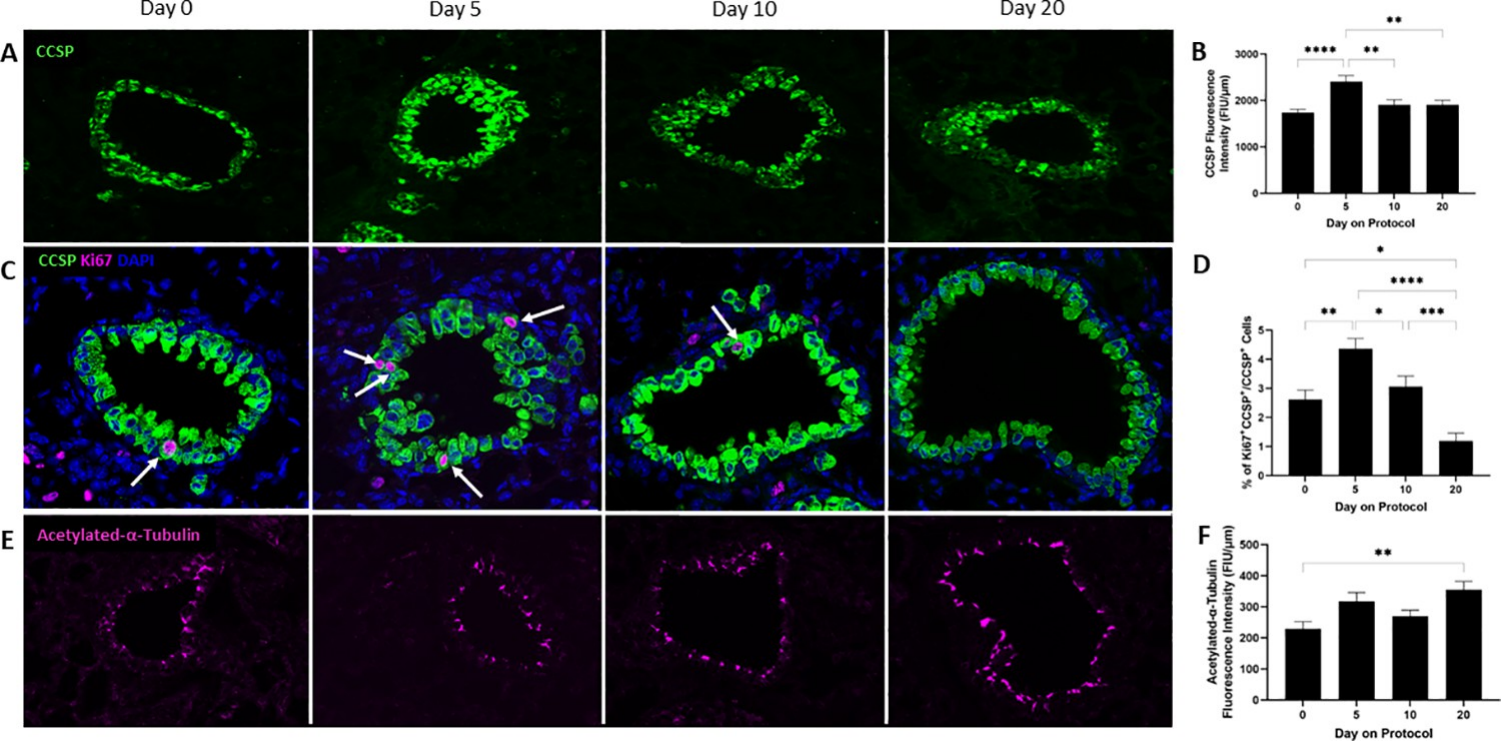
SO_2_ exposure triggers proliferation and differentiation of club cells. (A, C, and E) Representative images of SA from lung sections of mice at the indicated protocol time points stained using immunofluorescent antibodies targeting club cell secretory protein (CCSP, A and C), Ki67 (C), DAPI (C), or/and acetylated-α-tubulin (E). Note the abundance of CCSP^+^ cells in the airway of SO_2_-exposed mice at protocol day 5 suggestive of club cell hyperplasia. (B and F) Fluorescent intensity of CCSP (B) and acetylated-α-tubulin (F) within the small airway epithelium was quantified and normalized to the length of the subepithelial basement membrane. (D) The percentage of proliferating club cells [(Ki67^+^DAPI^+^CCSP^+^/DAPI^+^CCSP^+^)*100] in each SA was determined. Data represent mean ± SEM of 89–100 SA; ten randomly selected or all SA per lung lobe, 4–5 lobes per mouse, two mice per time point. * P < 0.05, ** P < 0.01, **** P < 0.0001; one-way ANOVA followed by Tukey’s multiple comparisons test.

As both SO_2_ exposure (in this study) and sustained club cell injury (in our prior study [23]) led to the development of peribronchiolar fibrosis, we assessed the effects of SO_2_ exposure on airway club cells. Under homeostatic conditions, the turnover of epithelial cells lining the SA is low [30]. In response to injury, however, club cells that serve as local progenitors proliferate rapidly to repair structural damage. Following proliferation, club cells slowly differentiate into ciliated cells over the course of several weeks. To study the impact of SO_2_ exposure on small airway epithelial integrity, lung sections of mice at protocol day 0 (unexposed), 5, 10, and 20 were stained using immunofluorescence for CCSP and acetylated-α-tubulin to mark club and ciliated cells, respectively. Representative fluorescent microscopy images of SA from these sections are shown in Fig 8A and 8E. By visual inspection, epithelial hyperplasia was identified at protocol day 5 as evidenced by an apparent increase in cells staining positive for CCSP. Hyperplasia appeared diminished at protocol day 10 and 20. To assess this further, fluorescence intensity of CCSP and acetylated-α-tubulin in the epithelium of SA was quantified and normalized to the length of the subepithelial basement membrane. Using this technique, a transitory increase of more than 38% in normalized CCSP fluorescence intensity was detected at protocol day 5 versus day 0 (Fig 8B) suggesting that SO_2_ exposure induces small airway epithelial injury that triggers club cell proliferation.

To confirm SO_2_ exposure-induced club cell proliferation, rather than mere enhanced CCSP expression within club cells, lung sections of mice at protocol day 0 (unexposed), 5, 10, and 20 were stained using immunofluorescence for the proliferation marker Ki67, CCSP, and DAPI (Fig 8C). Quantifying the percentage of proliferating club cells in the epithelium of SA showed a transient 67% increase in lungs of mice from protocol day 5 versus day 0 (Fig 8D).

To determine whether proliferating club cells might be repopulating injured ciliated cells, the fluorescence intensity of acetylated-α-tubulin was quantified and revealed an increase of 54% in normalized acetylated-α-tubulin fluorescence intensity at protocol day 20 versus day 0 (Fig 8F), likely indicating differentiation of club cells into ciliated cells. Collectively, we conclude that a repetitive, 5-day exposure of mice to SO_2_ causes the defining feature of constrictive bronchiolitis, namely subepithelial collagen deposition leading to peribronchiolar fibrosis, and we implicate epithelial injury followed by local club cell proliferation in the pathogenesis of this condition.

## Discussion

The results of this study demonstrate that exposure of mice to 50 ± 5 ppm of SO_2_ for one hour a day for five consecutive days is sufficient to mediate substantial injury to the epithelium lining SA in the lung as evidenced by local progenitor club cells triggered to exit their normal, quiescent state and undergo robust proliferation and differentiation. Moreover, our data show that resolution of this small airway injury is abnormal and leads to subepithelial collagen deposition culminating in increased small airway wall thickness, which is the defining histopathologic feature of DRCB. Lack of commonly observed, associated histopathological features of DRCB such as peribronchiolar immune infiltrates and respiratory bronchiolitis in mice exposed to SO_2_ using the current protocol suggests that exposure to additional airborne hazards may contribute to the development of this disorder.

Clinically relevant animal models to study the pathogenesis of DRCB and to identify potential biomarkers, advanced imaging modalities, treatment strategies, and monitoring techniques for this disease are much needed. Yet modelling this condition is challenging due to many uncertainties about the specific type, dose, and duration of exposure to inhalational toxins that might cause DRCB. To circumvent this problem, we previously presumed that many inhalational toxins may injure airway club cells and that sustained club cell injury may simulate a common injury pathway downstream of various airborne hazards exposure. We therefore established a club cell injury-induced murine model that was found to recapitulate many of the histopathological features detected in MP with DRCB [23]. The objective of the current study was to determine whether a specific exposure to SO_2_ could mimic the abnormalities observed in MP with DRCB and to compare SO_2_ exposed mice with doxycycline exposed CC-DTA mice that develop constrictive bronchiolitis in response to sustained club cell injury.

The central finding of the current study is that a repetitive, relatively brief SO_2_ exposure of mice is sufficient to induce peribronchiolar fibrosis which is the defining feature of DRCB. We used an SO_2_ concentration of 50 ppm in this study to match the estimated SO_2_ concentration near the perimeter of the Al-Mishraq sulfur plant fire [20] In addition, brief exposures to this SO_2_ concentration was reported to enhance subepithelial fibrosis in SA of mice repeatedly challenged with ovalbumin [32]. The peribronchiolar fibrosis we observed in mice in response to SO_2_ exposure recapitulated findings described by King et al [14] in MP diagnosed with DRCB at Vanderbilt University. Amongst those diagnosed, 74% reported exposure to the 2003 fire at the sulfur enrichment facility near Mosul, Iraq. Gutor et al [16] recently published a more detailed histopathologic analysis of surgical lung biopsies from MP diagnosed with DRCB at the same institution. Using PSR staining of lung sections and morphometric measurements, they identified increased subepithelial collagen content in SA of subjects with DRCB relative to non-diseased controls that was associated with small airway wall thickening. Although we lacked sufficient lung biopsy samples to perform a comparable analysis at our institution, we demonstrate here that the peribronchiolar fibrosis induced in mice by SO_2_ exposure is similar to that observed in MP diagnosed with DRCB using representative images of lung biopsies performed at our institution on four Veterans exposed to SO_2_ and other airborne toxins.

Our study design differs from other reports of SO_2_ exposure in animal models. For example, Wigenstam et al [33] exposed rats to a single dose of 2200 ppm SO_2_ for 10 minutes. Severe damage restricted to the larger bronchi was detected within hours post exposure and consisted of widespread mucosal erosion and ulceration of the epithelium which was associated with transient inflammation. Interestingly, increased airway expression of TGFβ1 and total lung collagen content were detected suggesting that this high intensity SO_2_ exposure might have led to lung fibrosis although quantitative morphometric analysis was not performed to assess the microanatomic location of collagen deposition. These findings suggest that exposure to a single, short, very high concentration of SO_2_ mediates pulmonary effects that differ from those observed after repeated, low to moderate doses of SO_2_ exposures.

SO_2_ toxicity has been attributed, at least in part, to its capacity to induce oxidative stress [34, 35], however, specific cellular targets within the epithelium lining the SA critical to inhalational injury haven’t been fully identified. Peribronchiolar fibrosis mediated by SO_2_ exposure in this study was comparable to that described in CC-DTA mice subjected to targeted, sustained club cell injury [22, 23]. Furthermore, alterations in serum CCSP levels were detected in SO_2_-exposed workers operating a non-ferrous smelter [36]. This led us to study the effects of SO_2_ exposure on airway club cells. We identified club cell hyperplasia immediately following the 5-day SO_2_ exposure protocol. We further identified evidence of increased acetylated-α-tubulin fluorescence intensity in SA at protocol day 20 suggesting that club cells differentiated into ciliated cells in response to SO_2_-induced injury. Thus, in both mouse models, development of peribronchiolar fibrosis was associated with club cell injury and proliferation. Club cell hyperplasia and peribronchiolar fibrosis in response to repetitive SO_2_ exposures (5 hours/day, 5 days/week for 23 or 43 days at 400 ppm) were reported by Morgenroth et al [37] even though morphometric analysis was not performed. Our findings parallel an evolving paradigm identifying injury to progenitor type 2 alveolar epithelial cells as a stimulus for the development of subepithelial fibrosis in the alveolar compartment [38]. It is possible that SO_2_-induced proliferating CCSP^+^ cells observed in this study contain recently described bronchoalveolar stem cells that express both CCSP and cytokeratin 8 (CCSP^+^Krt8^+^) as this progenitor population has been implicated in the development of alveolar fibrosis in mice exposed to bleomycin and in patients with fibrotic lung disease [39, 40]. This intriguing hypothesis warrants further exploration in future lineage tracing studies. Collectively, these findings implicate SO_2_ injury to the small airway epithelium and particularly to club cells in the pathogenesis of constrictive bronchiolitis. Even though mouse models have been instrumental to our understanding of human lung diseases, it should be noted that anatomical, histological, and physiological differences between mouse and human lung may affect the host response to inhalational small airway injury [41–44].

Morphometric/quantitative analysis of murine lung sections were used in this study to advance our understanding of molecular processes underlying SA subepithelial collagen deposition leading to airway wall thickening and peribronchiolar fibrosis. Using Masson’s trichrome staining to identify collagen, our data first demonstrated that SO_2_ exposure resulted in thickening of small airway walls as measured by the normalized area of collagen deposition surrounding the SA. We validated and extended these findings using analysis of lung sections stained with PSR. PSR is a histological dye used for staining collagen in tissue sections as the elongated, anionic Sirius red molecule binds parallel to cationic collagen fibers. Fluorescent imaging of PSR-stained tissue sections elicits a strong, red fluorescence signal that is sensitive and specific for collagen and thus has recently emerged as a useful technique for visualization [16] and quantitative analysis [27] of collagen deposited in tissue samples. PSR staining has been shown to detect both fibrillar (type I) and non-fibrillar (type IV) collagens [27], which might explain the temporal discrepancies we observed when analyzing Masson’s trichrome- versus PSR-stained lung sections in this study. CurveAlign [24, 25] and CT-FIRE [25–27], collagen fiber extraction software available as an open-source on MATLAB, are powerful quantitative tools recently designed to analyze collagen structure and architecture within cancer tissues in order to explore the potential role of collagen in cancer progression and invasion [45]. CurveAlign was developed for bulk assessment of collagen features including number, density, and alignment of fibers, whereas CT-FIRE was designed for quantification of individual fiber metrics such as width and length. Although CurveAlign has been recently employed for in vitro studies of fibroblast migration dynamics in idiopathic pulmonary fibrosis [46], we believe that this is the first report to use these tools to analyze collagen content in the wall of fibrotic SA. Here, our CurveAlign and CT-FIRE-based findings suggest that the molecular mechanisms responsible for the development of peribronchiolar fibrosis in response to SO_2_ exposure involve an increase in the width, length, and number, but not density, of collagen fibers in the walls of SA. Due to the rigor required to perform quantitative morphometric analysis, a limited number of mice were assessed at each time point which may be a limitation of this study. We believe this model might prove useful in further enhancing our understanding of how individual collagen fibers within the wall of SA gradually thicken and eventually elongate while additional fibers are deposited de novo.

We did not identify prominent features of airway inflammation in response to this SO_2_ exposure protocol. This observation is consistent with reported lack of airway inflammation in mice exposed to a single dose of 100 ppm SO_2_ for 4 hours [47] yet contrasts with frequent findings of chronic inflammation in SA of MP diagnosed with DRCB [14–16] and with peribronchiolar immune infiltrates and airway macrophage accumulation described in sustained club cell injury-induced murine constrictive bronchiolitis [23]. In their recent follow-up study, Gutor et al [48] profiled immune and inflammatory gene expression in lung sections obtained from MP with DRCB and reported T cell activation suggesting that a chronic adaptive immune response may be involved in the pathogenesis of this disease. Since adaptive immune responses develop more slowly, it’s possible that the SO_2_ exposure protocol employed in this study was incapable of stimulating a discernable T cell response. Alternatively, or in addition, induction of T cell responses in DRCB may require the presence of an antigenic stimuli or another form of immune perturbation. Exploring these hypotheses will be important yet exceeds the scope of the current study. Altogether, it is possible, and even likely, that multiple exposures to airborne hazards, particularly those involving high concentrations of particulate matter or any form of oxidative gas, contribute to the development of DRCB.

In summary, our findings implicate SO_2_, as at least one of the deployment-related airborne hazard exposures, capable of causing small airway epithelial injury leading to the development of peribronchiolar fibrosis. Although not all MP diagnosed with DRCB were exposed to the fire at the Al-Mishraq sulfur enrichment facility in 2003, other exposures to SO_2_ during deployment were prevalent as it was a component of burn pit smoke and likely a regional industrial pollutant. SO_2_ exposure induces alterations in airway club cell populations further extending our prior study which identified club cells as central to CB pathogenesis. The National Heart, Lung, Blood Institute now advocates use of more than one animal model when studying pathogenesis, biomarkers, or treatment of fibrotic interstitial lung diseases. Our study suggests that ongoing use of this SO_2_ exposure model in mice will yield additional insight that may advance our understanding of fibrotic small airway diseases including DRCB.

## Supporting information

S1 Fig Peribronchiolar fibrosis in deployment-related respiratory disease. Representative images of SA from H&E- (A and C) and Masson’s trichrome- (B and D) stained sections of surgical lung biopsies of two Veterans diagnosed with deployment-related respiratory disease. Note thickening of the airway wall due to subepithelial collagen deposition marked by black arrows. Magnification: x300 (A), x200 (B), x100 (C and D).
(TIF)

S2 Fig Implementation of gating strategy for identifying lung myeloid subsets in control mice. (A-I) Lung-derived single cell suspensions obtained from control mice were stained using fluorochrome-conjugated antibodies targeting CD45, Ly6G, CD11b, Siglec F, CD11c, Ly6C, CD24, CD103, and MHC class II and analyzed by flow cytometry. Representative dot plots from a single control mouse evaluated on protocol day 10 are shown. After excluding doublets, debris, and dead cells, CD45+ white blood cells were identified (A). Neutrophils were detected based on expression of Ly6G and CD11b (B). Non-neutrophils, Siglec F+ cells (C) were depicted on CD11b versus CD11c plots to identify eosinophils and alveolar macrophages (AM, D). Ly6C+ monocytes were defined as non-neutrophils, Siglec F-CD11c-CD11b+Ly6C+ cells (E and F). Conventional DC (cDC) were identified as non-neutrophils, Siglec F-CD11c+CD24+ cells (G) and further classified based on expression of CD103 or CD11b (H). CD24- cells within the CD11c+ gate were depicted on MHC class II versus autofluorescence (detected in the FITC channel) plots to identify exudate macrophages (EM) and monocyte-derived DC (mDC, I). FSC, forward scatter; SSC, side scatter; A, area; DC, dendritic cells, MHC, major histocompatibility complex.
(TIF)

S3 Fig Peribronchiolar inflammation and respiratory bronchiolitis in deployment-related respiratory disease. Representative images of SA from lung sections of two Veterans diagnosed with deployment-related respiratory disease. Panels A, B, and D were stained with H&E; C panel was stained for CD68 using immunohistochemistry (brown stain depicts monocytes and macrophages). Note peribronchiolar mononuclear cell infiltrates (A and B), intraluminal collection of CD68^+^ cells (C), and clusters of intraluminal cells resembling big, foamy macrophages (D). AW, airway.
(TIF)

S1 Table Antibodies used for flow cytometry analysis. PerCP-Cy5.5, peridinin chlorophyll protein-cyanine 5.5; APC, allophycocyanin; APC-Cy7, allophycocyanin-cyanine 7; PE-CF594, phycoerythrin-CF594; PE, phycoerythrin; PE-Cy5, phycoerythrin-cyanine 5; PE-Cy7, phycoerythrin-cyanine 7; FITC, fluorescein isothiocyanate.
(PDF)

S1 Raw data (XLSX)
