# Supplementary figures and images for: Sulfur dioxide exposure of mice induces peribronchiolar fibrosis—A defining feature of deployment-related constrictive bronchiolitis
Source: PLoS One. 2025 Jan 24;20(1):e0313992. doi: 10.1371/journal.pone.0313992 (PMC11761160; doi:10.1371/journal.pone.0313992)

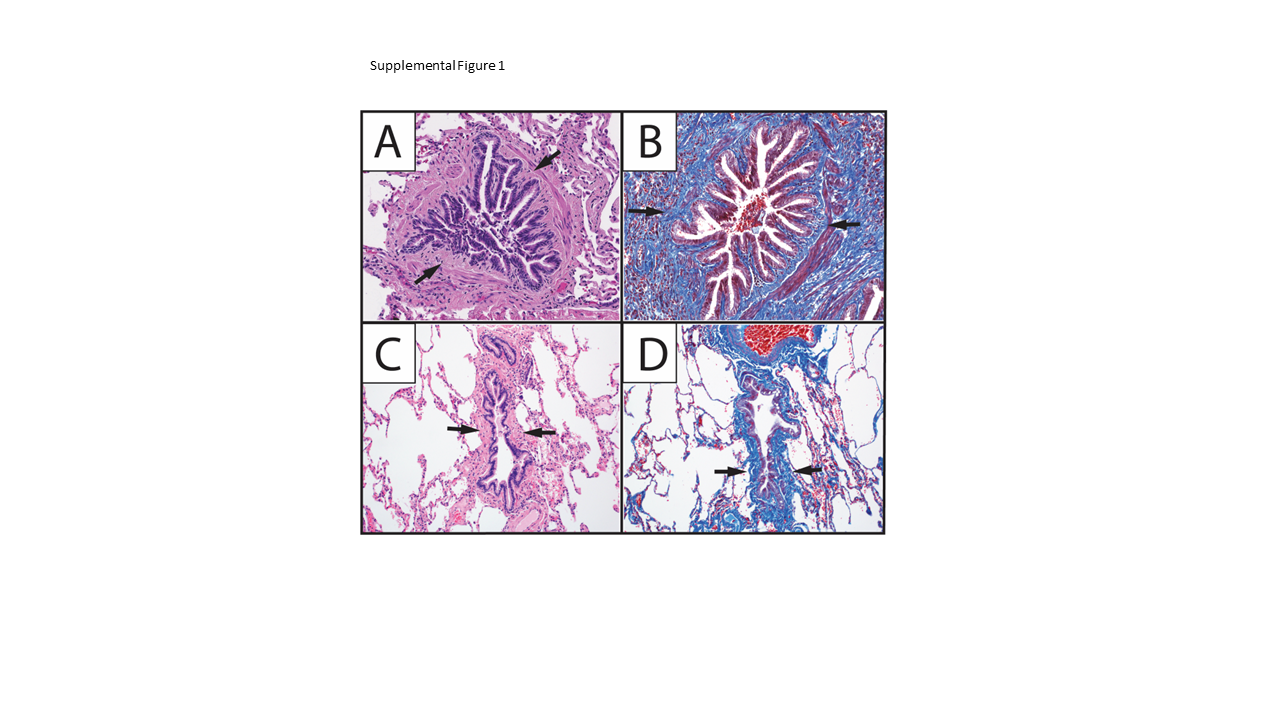

Supplement: S1 Fig — Representative images of SA from H&E- (A and C) and Masson’s trichrome- (B and D) stained sections of surgical lung biopsies of two Veterans diagnosed with deployment-related respiratory disease. Note thickening of the airway wall due to subepithelial collagen deposition marked by black arrows. Magnification: x300 (A), x200 (B), x100 (C and D). (TIF) [file pone.0313992.s001.tif]

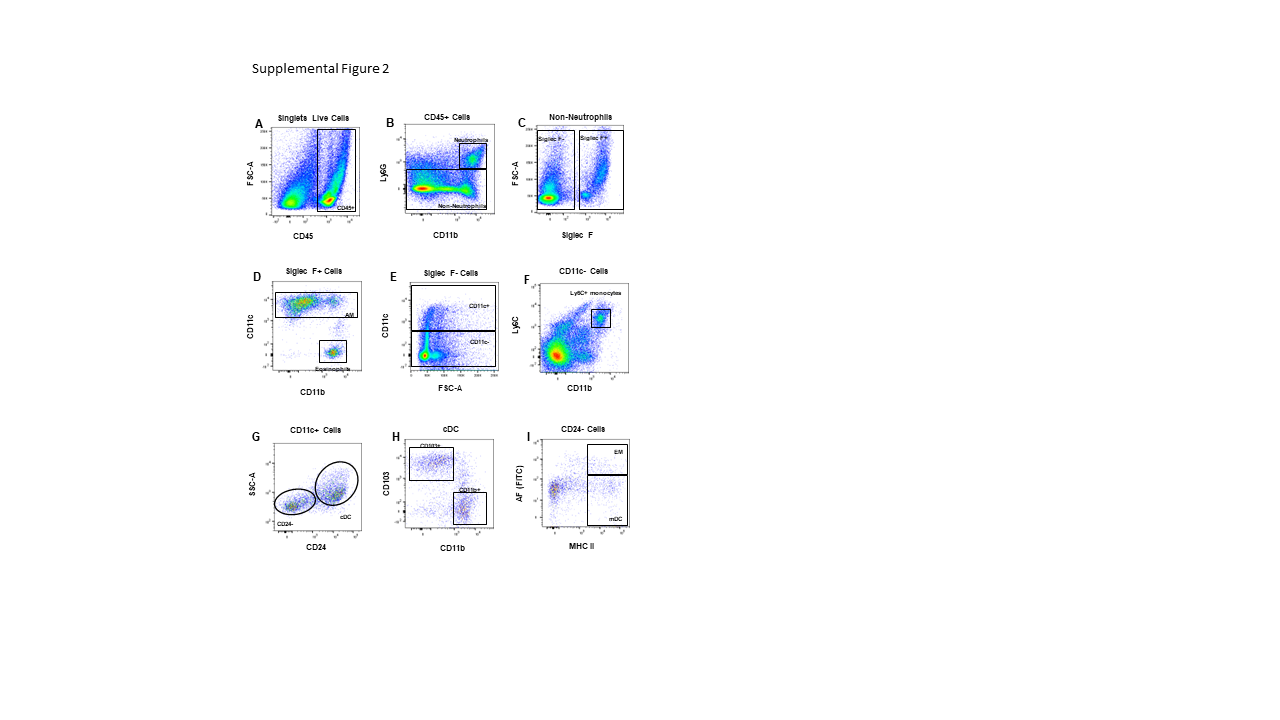

Supplement: S2 Fig — (A-I) Lung-derived single cell suspensions obtained from control mice were stained using fluorochrome-conjugated antibodies targeting CD45, Ly6G, CD11b, Siglec F, CD11c, Ly6C, CD24, CD103, and MHC class II and analyzed by flow cytometry. Representative dot plots from a single control mouse evaluated on protocol day 10 are shown. After excluding doublets, debris, and dead cells, CD45+ white blood cells were identified (A). Neutrophils were detected based on expression of Ly6G and CD11b (B). Non-neutrophils, Siglec F+ cells (C) were depicted on CD11b versus CD11c plots to identify eosinophils and alveolar macrophages (AM, D). Ly6C+ monocytes were defined as non-neutrophils, Siglec F-CD11c-CD11b+Ly6C+ cells (E and F). Conventional DC (cDC) were identified as non-neutrophils, Siglec F-CD11c+CD24+ cells (G) and further classified based on expression of CD103 or CD11b (H). CD24- cells within the CD11c+ gate were depicted on MHC class II versus autofluorescence (detected in the FITC channel) plots to identify exudate macrophages (EM) and monocyte-derived DC (mDC, I). FSC, forward scatter; SSC, side scatter; A, area; DC, dendritic cells, MHC, major histocompatibility complex. (TIF) [file pone.0313992.s002.tif]

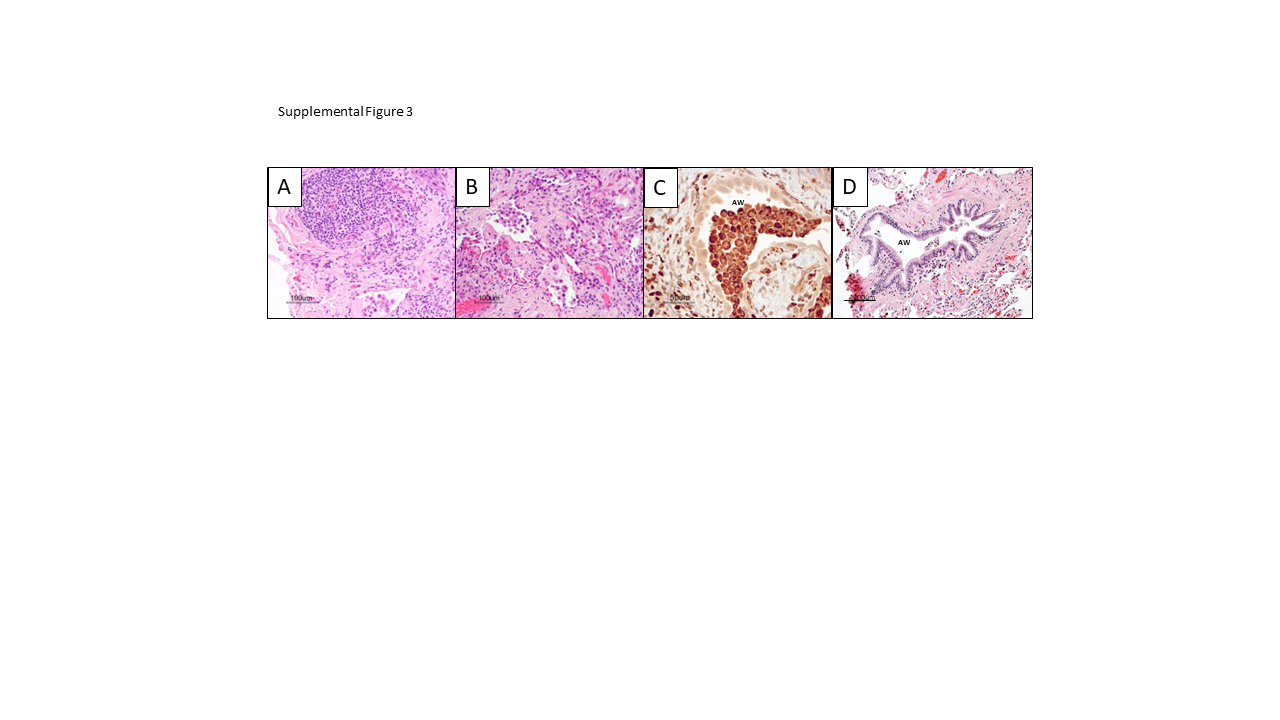

Supplement: S3 Fig — Representative images of SA from lung sections of two Veterans diagnosed with deployment-related respiratory disease. Panels A, B, and D were stained with H&E; C panel was stained for CD68 using immunohistochemistry (brown stain depicts monocytes and macrophages). Note peribronchiolar mononuclear cell infiltrates (A and B), intraluminal collection of CD68+ cells (C), and clusters of intraluminal cells resembling big, foamy macrophages (D). AW, airway. (TIF) [file pone.0313992.s003.tif]
