# Supplementary material for: Sulfur dioxide exposure of mice induces peribronchiolar fibrosis—A defining feature of deployment-related constrictive bronchiolitis
Source: PLoS One. 2025 Jan 24;20(1):e0313992. doi: 10.1371/journal.pone.0313992 (PMC11761160; doi:10.1371/journal.pone.0313992)
Supplement: S1 Table — PerCP-Cy5.5, peridinin chlorophyll protein-cyanine 5.5; APC, allophycocyanin; APC-Cy7, allophycocyanin-cyanine 7; PE-CF594, phycoerythrin-CF594; PE, phycoerythrin; PE-Cy5, phycoerythrin-cyanine 5; PE-Cy7, phycoerythrin-cyanine 7; FITC, fluorescein isothiocyanate. (PDF) [file pone.0313992.s004.pdf]

S1 Table

S1 Table. Antibodies used for flow cytometry analysis

Target	Clone	Fluorochrome	Manufacturer
CD45	30-F11	PerCP-Cy5.5	BioLegend
Ly6G	1A8	APC	BioLegend
CD11b	M1/70	APC-Cy7	BioLegend
CD11c	N418	Brilliant Violet 421	BioLegend
Siglec F	E50-2440	PE-CF594	BD Biosciences
CD24	M1/69	Brilliant Violet 650	BD Biosciences
CD103	2E7	PE	BioLegend
MHC II (I-A/I-E)	M5/114.15.2	PE-Cy5	BioLegend
Ly6C	HK1.4	Alexa Fluor 700	BioLegend
CD206	C068C2	PE-Cy7	BioLegend
TCRβ chain	H57-597	PE-Cy5	BioLegend
CD8a	53-6.7	FITC	BioLegend
CD4	GK1.5	APC	BioLegend
CD19	1D3	PE-CF594	BD Biosciences

PerCP-Cy5.5, peridinin chlorophyll protein-cyanine 5.5; APC, allophycocyanin; APC-Cy7, allophycocyanin-cyanine 7; PE-CF594, phycoerythrin-CF594; PE, phycoerythrin; PE-Cy5, phycoerythrin-cyanine 5; PE-Cy7, phycoerythrin-cyanine 7; FITC, fluorescein isothiocyanate.
